# Response of Connections between Concrete Corbels and Safety Barriers

**DOI:** 10.3390/ma12244103

**Published:** 2019-12-08

**Authors:** Sara Cattaneo, Pietro Crespi

**Affiliations:** 1 Department of Architecture, Built Environment and Construction Engineering, Politecnico di Milano, Piazza Leonardo da Vinci 32, 20133 Milano, Italy; pietro.crespi@polimi.it; 2 Construction Technologies Institute, Italian National Research Council (ITC-CNR), Viale Lombardia 49, San Giuliano Milanese, 20098 MI, Italy

**Keywords:** post-installed bonded anchors, concrete corbel, bridge deck, safety barrier anchoring

## Abstract

Post-installed anchor systems are widely used in several applications both for retrofit and new constructions due to their flexibility and easiness of installation. For these reasons, their use is very common to connect safety barriers to concrete corbels. However, the design requirements for fastening (Eurocode 2—Part 4, ACI 318 or for post-installed rebar (Eurocode 2-Part 1 can be hardly satisfied due to the geometric restrictions of the application. Additional complications arise for the refurbishment of bridge corbels, which usually requires the removal of the damaged top concrete layer. This paper presents a new anchoring system that consists of the use of additional post-installed U-shaped rebars connecting the lower existing portion of the corbel to the upper (rebuilt) layer, able to carry the required design loads. The proposed solution considers three anchors that transfer the external loads to the corbel, to the existing reinforcements, and to the U-shaped rebars. The system is tested experimentally and validated by using both theoretical (strut and tie modeling) and numerical analyses.

## 1. Introduction

Post-installed anchor systems are very flexible and useful in different critical situations [1,2], nevertheless in some cases their use is limited (i.e., geometrical restrictions, mechanical properties of the base material, lack of design guidelines). To increase the range of applications and to improve the safety level, several researches have been performed in the last years [2,3,4,5,6,7,8,9,10,11,12,13] exploring both the range of applicability (i.e., considering high/low strength concrete [3,4,5,6] or reduced geometry [7]), investigating the safety level of existing codes [8,9], and the reliability of the connections. This last aspect has been investigated with particular reference to adhesive anchors [10,11,12,13] since the in-situ conditions could strongly affect the anchorage performance and the safety level as well. Nevertheless, if properly installed, post-installed anchors are highly reliable and very popular both to strengthen existing structures and to connect existing concrete elements to new ones.

In the last decades, the refurbishment of existing buildings and infrastructures has become very important due to their ageing or lack of maintenance [2,14,15]. A similar problem is the substitution/installation of safety barriers in existing bridges over their service life. The safety barrier substitution is usually associated with the partial reconstruction of the concrete corbel and with the addition of post-installed bonded systems, which provide high flexibility and strength—sometimes well beyond cast-in anchor solutions [16,17]. Unfortunately, in many cases, this kind of connection does not satisfy code requirements for the design of the connectors considered as overlapped rebars [18] or as post-installed anchors [18,19,20,21,22,23]. Indeed, the limited thickness of the concrete corbel often does not guarantee a sufficient bonded length, and the edge distance does not allow the full development of the concrete capacity if anchor theory [1] is adopted. Other solutions for safety barriers anchorage have been proposed considering post-installed mechanical anchors [24], but these other configurations are rather uncommon in Italy.

This study proposes a new solution, based on the removal and rebuilding of the damaged top concrete layer of the corbel, which meets the common refurbishment practice in Italy (with slight modifications). The novelty of the proposed intervention consists in the installation of additional U-shaped post-installed bonded rebars, with a prescribed spacing along the whole corbel length. The rebars are placed close to the tensioned anchors position, before the new top concrete layer is cast. Safety barriers are then installed onto the new reinforced corbel via three post-installed bonded bars. The aim of the additional rebars is to increase the tension load that can be applied to the post-installed bars by improving the degree of connection between the new upper corbel and the lower slab.

To evaluate the influence of the U-shaped rebars in the connection system, three full scale laboratory specimens were tested to check the effectiveness of the solution. Although the actual applied load to safety barriers is an impact load [24], the experimental investigation was based on quasi-static tests according to Italian standard [25] (which prescribes to apply the load that induce the failure of the post with a safety factor equal to 1.5). This choice was considered to provide conservative results based on the fact that impact tests are affected by the stiffness of the barrier (out of the scope of this research) and that, as it is well known, the static capacity of the anchorage is expected to be lower than its dynamic capacity [24,26,27,28]. Although the whole corbel behavior should be properly analyzed [29], this study focuses on the transfer mechanism of the forces from the tensioned bars to the U-shaped rebars and their anchorage into the bridge deck. For this purpose, a 3D strut and tie model was developed to evaluate the forces acting on the U-shaped rebars and to optimize the connection. Finally, a non-linear numerical analysis was performed to validate the theoretical model. The proposed approach could be also adopted in other applications where geometrical restrictions influence the ability of the connection to carry the design loads.

## 2. Experimental Investigation

### 2.1. Sample Preparation

**Figure 1:**
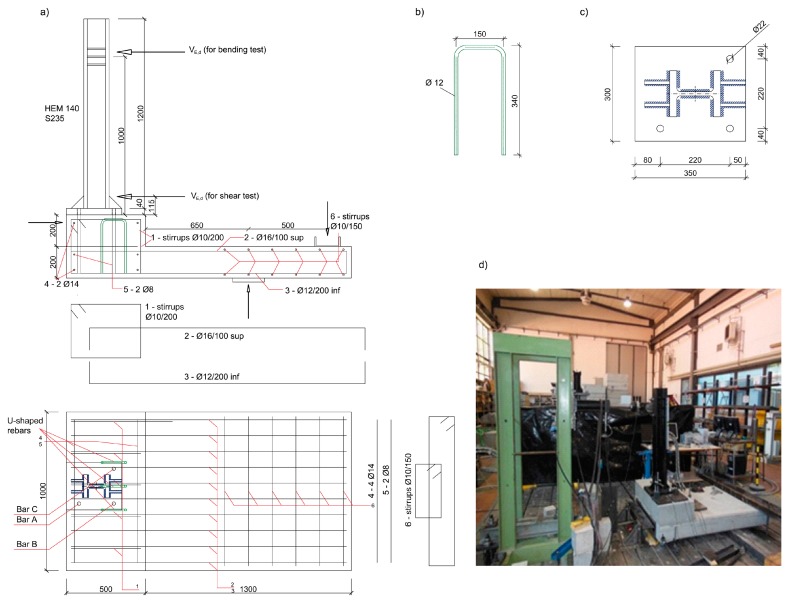
(**a**) Specimen and test set-up, (**b**) U-shaped rebar, (**c**) steel plate and (**d**) test set-up (measures in mm).

Three identical reinforced concrete specimens made of a slab and a corbel were realized, having dimensions 180 cm × 100 cm × 20 cm and 50 cm × 100 cm × 20 cm respectively (Figure 1a). The corbel reinforcement was chosen as a typical low reinforcement of Italian bridge corbels, while the slab reinforcement was designed to avoid any possible anticipated failure during the tests. The samples were cast in two steps using C20/25 concrete and B450C rebars. The concrete mix-design was defined according to the requirements reported in [30], for an average cubic (side 150 mm) compressive strength ranging between 31.6 and 32.2 MPa, according to EN 12390-3 [31]. The yielding strength of rebars was 512 MPa.

The slab was cast first and, after 28 days, three ∅12 U-shaped B450C rebars were installed using an epoxy injection mortar with a characteristic bond strength of 14 MPa [32]. The U-shaped rebars were installed protruding outside the upper face of the former slab by 170 mm. After 24 h, the corbel was cast above the slab and, after additional 28 days, three M20 bars (B500) were post-installed in the slab through the corbel. The slab was not treated (roughened) in order to have a bad cold joint and to reproduce the worst condition available in jobsite.

The geometry of the U-shaped rebars is shown in Figure 1b. Three ∅12 rebars were installed in drilled holes with diameter of 16 mm and effective embedment depth in the slab of 170 mm with a spacing (center-to-center) of 150 mm. The M20 bars were spaced 220 mm (Figure 1c) and installed in holes with a diameter of 25 mm with an effective embedment depth of 370 mm (Table 1), crossing the new corbel and the former slab. Before anchor installation, holes were cleaned according to the product’s MPII (manufacturer’s product installation instruction [32]). A tightening torque T_inst_ = 150 Nm was applied. The diameter of the hole in the steel fixture was 22 mm. The final test setup is shown in Figure 1a,d.

The load V_E_ was applied by a hydraulic jack via a steel cantilever at a height of 100 cm from the steel base plate (Figure 1a–d). The concrete specimen was supported in the middle of the slab and vertically restrained at a distance of 50 cm from the support. Additionally, a horizontal restraint was placed in front of the specimen to prevent its sliding. The vertical displacements of the three M20 bars were measured via LVDTs (HBM, Darmstadt, DE). The applied load was measured by a load cell and all data were acquired with a HBM Spider 8 device with a sampling rate of 2 Hz.

The crack width of the main cracks was measured with a crack width microscope (magnification 40× up to 4 mm) or with a caliper (width larger than 4 mm).

### 2.2. Test Protocol

The tests were displacement controlled at a constant rate of 0.05 mm/s. The load protocol was:

- Three loading cycles from 0 kN to 38 kN;
- Loading up to 51 kN and check of crack pattern (load held for 2–3 min);
- Loading up to 65 kN for tests 1 and 2 while up to 84 kN for test 3, to inquire further capacity of the specimen;
- Unloading.

According to the Italian Standard [25], a load of 51 kN was chosen as the ultimate limit state design load V_E,d_ and the previously described geometry of the specimen was designed according to this load. Note that, for exceptional actions, the partial safety factor is 1 for both materials and actions. The three cycles were performed at about 75% of the design load, while the maximum load of the first two tests was chosen to increase of about 30% the design load by keeping the loads acting on the posts into their elastic range. The third test was stopped at a load level of 84.07 kN (65% more than V_E,d_) to avoid damage in the test equipment.

After completion of the loading protocol, the third specimen was subjected to an additional test to evaluate its residual shear capacity. In this test, the horizontal load was applied at 11.5 cm above the concrete top surface via a hydraulic jack with a load capability of 300 kN.

## 3. Experimental Results

No failure was observed during the tests. All specimens behaved in a similar way, showing a bearing capacity well beyond the design load (even more than 30%). At a load level of about 47 kN, two cracks (one for each lateral side) appeared at the corner between the slab and the corbel as shown in Figure 3 for specimen #1. All specimens exhibited the same cracks, which reached a width of about 0.16 mm for specimen #1 and 2 at the maximum load level (about 65 kN) and of about 0.18 mm for specimen #3 (about 84 kN). When the specimens were unloaded the two cracks closed and just hairline cracks were visible. In specimens #2 and #3, at a load of about 65–68 kN, additional cracks (see Figure 3) were observed originating from the most external bar and developing continuously up to the reached maximum load (about 84 kN). The main information regarding the crack patterns observed after the tests are summarized in Figure 3. In specimen #2 the maximum crack width at the peak load was 0.5 mm, while in specimen #3 the maximum crack width was 0.73 mm.

The results in terms of load-displacement curves of the specimen #3 are reported in Figure 4 (Specimen #1 and #2 exhibited a similar behavior showing a good repeatability of the results). In particular, the load vs. vertical displacement curves of the three M20 bars are plotted in Figure 4a, while a focus on the first three cycles up to 38 kN is shown in Figure 4b.

At the end of the test, specimen #3 was unloaded to allow the execution of a pure shear test to evaluate the residual shear capacity of the system. The shear test was stopped at a load of 200.9 kN to avoid potential damages to the testing equipment. During the test, some cracks developed and the rear bars were bent as shown in Figure 5. A concrete edge-like cone shaped failure surface developed from bar A. Nevertheless, the specimen was still able to carry the applied load although large cracks were detected (maximum crack width of 5 mm).

## 4. Discussion

**Figure 2:**
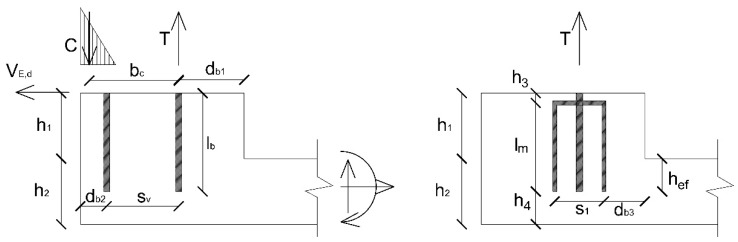
Geometry and applied loads on: (**left**) the corbel and (**right**) the additional U-shaped rebars.

**Figure 3:**
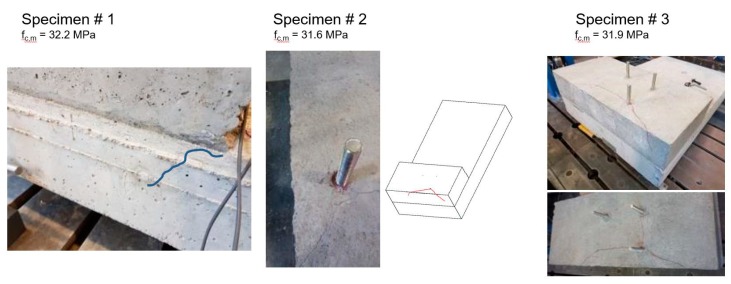
Crack patterns of tested specimens.

**Figure 4:**
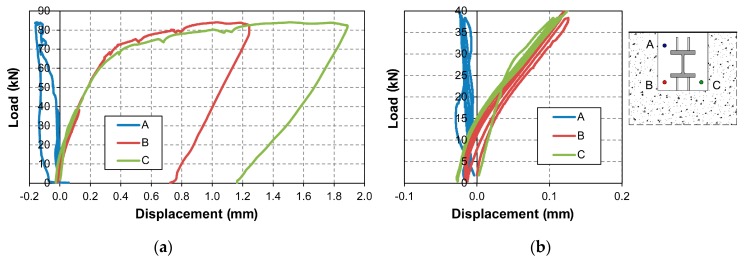
Specimen #3: load—anchor displacement curves: total (**a**) and detail (**b**).

**Figure 5:**
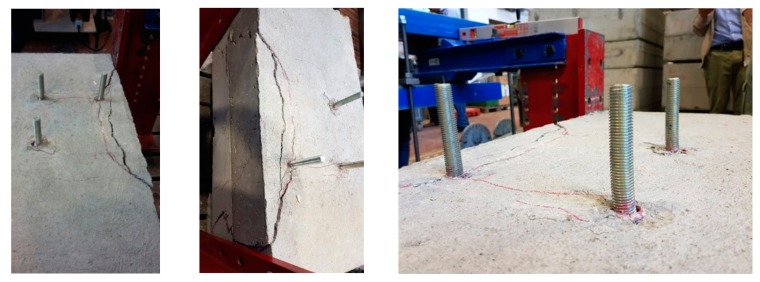
Specimen 3 after the shear test.

As shown in Figure 2, the application of a horizontal force V_E,d_ = 51 kN with a lever arm of 1 m, corresponding to the vertical distance between the connection and the application point of the force, resulted in a combined bending moment (M_E,d_ = 51 kNm) and shear load on the connection. By applying equilibrium and compatibility equations, the tensile force T_B_ (= T_C_) acting on a single bar of the connection can be calculated. The applied actions on the concrete corbel (Figure 2) could be summarized as follows:

- Tensile load T = 2T_B_ = 2∙93.32 kN ≅ 187 kN acting on the two rear bars (namely B and C according to the sketch reported in Figure 4);
- Compression load C = T on the concrete compressed surface;
- Shear load V_E,d_ acting on the front bar A (as shown in Figure 4).

A classical analysis, based on anchor theory, can easily show that both the two rear bars (due to concrete cone and splitting in tension) and the front bar (due to edge failure in shear) are not able to carry the applied tensile/shear loads (Figure 2) [1,19,20]. This happens because of the typical geometrical constraints (i.e., limited edge distance and embedment depth). Nevertheless, the experiments showed that the connection with post-installed bonded U-shaped rebars behaved properly and the carrying capacity of the system was well beyond the ultimate limit state (ULS). Therefore, to properly evaluate the capacity of the system, the U-shaped rebars must be accounted for.

The analysis of the geometry of the structural element suggests that the tensile load T is transferred from the two rear bars to the U-shaped rebars that are anchored in the lower part of the connection (i.e., concrete slab). The experimental evidence, as well as the anchor theory, showed that the weak point of the concrete corbel is the front anchor subjected to shear. Thus, a practical suggestion is to use a slotted hole in the steel base plate where the front bar A is placed in order to transfer the shear force only to the rear bars (B and C).

If this approach is adopted, the transfer mechanism of the load applied to the connection is expected to be as follows:

- The tensile load T applied to the two rear bars will be transferred to the concrete corbel and the additional U-shaped rebars;
- The shear load V_E,d_, applied to the two rear bars, will be transferred to the concrete corbel and to the transversal reinforcement (i.e., the existing stirrups);
- The compression load C generated by the steel plate will be spread into the concrete.

While the design of the corbel typically takes into account all acting forces, the focus of this study was solely on the transfer mechanism of the tensile load from the two rear bars (B and C) to the concrete corbel/U-shaped rebars. For the configuration under investigation, the tensile load T could be transferred mainly in two ways: overlapping or by concrete struts [33]. Although the former approach is simple and is valid in most practical applications, the latter seems to be more reliable due to the relative high distance (>4∅) between the bars and the U-shaped rebars. In the following, both approaches were considered. Firstly, a strut and tie model [18] was presented to verify the load transfer mechanism from the bars to the U-shaped rebars and, secondly, the anchorage of the U-shaped rebars in the slab was checked according to anchor theory.

### 4.1. Strut and Tie Model: Load Transfer to the U-Shaped Rebars

**Figure 6:**
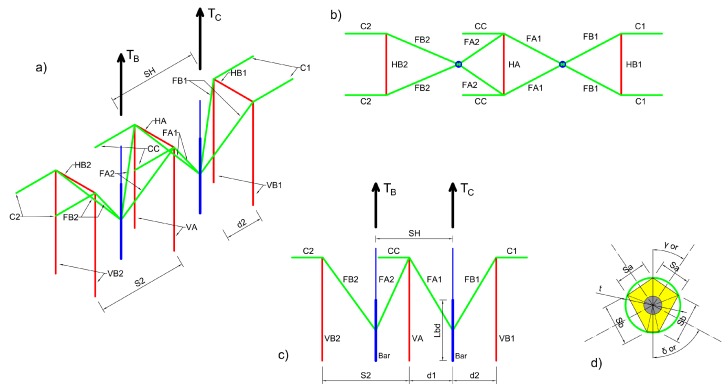
3D strut and tie model. (**a**) 3D view of the axis of the elements, (**b**) plan and (**c**) vertical view of the U-shaped rebars (red), concrete strut (green) and post-installed bars (blue) and (**d**) nodal region around the bar.

A 3D strut and tie model was chosen as the most appropriate approach to account for the actual geometry and complexity of the application. As shown in Figure 6, a force-transfer mechanism based on four compressed concrete struts connecting each M20 post-installed rebar to the corners of the adjacent U-shaped rebars could be considered.

While in the tests the U-shaped rebars were placed symmetrically with respect to the two post-installed bars, the model was developed to take into account variations in geometry and tolerances representative of actual job-site conditions. This follows the assumption that the three U-shaped rebars can be located in different positions with respect to the M20 post-installed bars, keeping the same center-to-center spacing (s_2_ = 150 mm, as for the tested configuration). This seems reasonable based on the fact that the installation of the U-shaped rebars is typically easier to be controlled and happens before the corbel is cast. To broaden the applicability of the model to real job-site conditions, the transfer length L_bd_ is also assumed as a parameter.

Each group of concrete struts converging to each bar is studied separately, since inclination angles can be different when symmetry is not available. Equilibrium conditions need to be verified taking into account also the horizontal components of the acting forces. Evaluation of the stresses in the concrete struts is necessary to define their geometry and cross-sectional area. Furthermore, the three-dimensional geometry of the node between the post-installed bar and the inclined concrete struts needs to be taken into account together with the dimensions of all the concrete struts, which depend on the radius of the circumscribed circle of the node’s cross section t (Figure 6d). Note that, given the minor importance of this parameter on the results of the model, its value can be conservatively fixed equal to 60 mm.

All the expressions of forces and stresses could be determined according to [34]. Since the critical aspect of the proposed solution is the verification of the U-shaped rebars and their anchorage into the concrete slab, the most relevant expressions are the ones describing the vertical forces in the U-shaped rebars (*V_B_*_1_, *V_A_* and *V_B_*_2_ in Equation (1)). As shown in Figure 6, the model allows the determination of the forces acting on the vertical legs of the U-shaped rebars as follows:

(1) $$V_{A}=\left[T_{B}\cdot \left(2\cdot s_{2}\;-\;s_{H}\right)\right]/\left(2\cdot s_{2}\right);\;V_{B1}=\frac{T_{B}\cdot \left(s_{2}\;-\;d_{2}\right)}{2\cdot s_{2}};\;V_{B2}=\frac{T_{B}\cdot \left(s_{H}\;-\;s_{2}\;+\;d_{2}\right)}{2\cdot s_{2}}.$$

For design purposes, all these actions must be lower than the maximum carrying capacity of the U-shaped rebars as follows:

(2) $$V_{i}\leq A_{S}f_{yd},$$

being *A_s_* and *f_yd_* the cross-section area of the U-shaped rebar and its yield strength, respectively.

**Figure 7:**
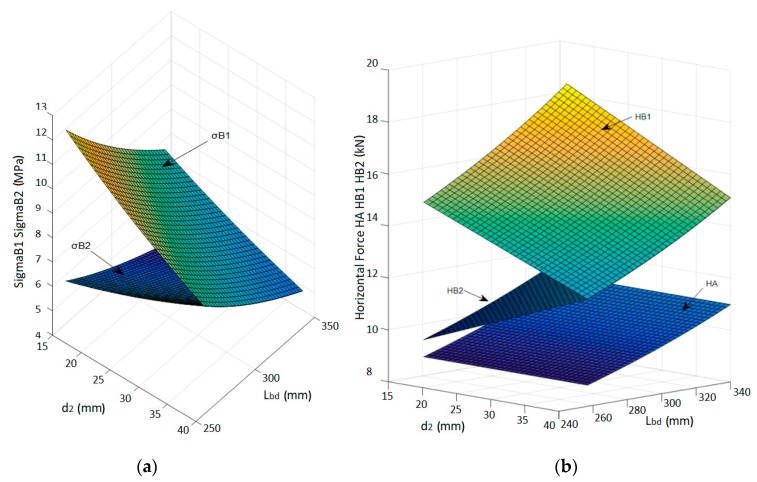
(**a**) Stresses in most stressed concrete struts and (**b**) forces on the horizontal arm of the U-shaped rebars (for: l_m_ = 340 mm, s_2_ =150 mm and t = 60 mm).

Other failure modes, as related to the failure of the concrete struts or to the failure of the horizontal arms of the rebars, were shown to not control the design. This is illustrated in Figure 7, where the stresses in the most stressed concrete struts (σ_B1_ and σ_B2_) and the tensile forces into the horizontal arm of the U-shaped rebars are plotted as a function of the distance d_2_ between bar and U-shaped rebar and as a function of the bar transfer length L_bd_.

The horizontal forces in the U-shaped rebars, which depend on both L_bd_ and d_2_, are always lower than 20 kN (it is worth noting that the tensile steel capacity of ∅12 rebars is equal to 50.84 kN).

The parametric study accounting for the influence of the transfer length and the relative distance between the U-shaped rebars and the post-installed bars highlighted that:

- The transfer length did not affect the vertical force in the U-shaped rebars and affected only the horizontal force, which increased with the transfer length. The design of the U-shaped rebars considered in this study (diameter 12 mm, B450C) was always satisfied for every position of the bars and for every practical value of the nodal region radius t. It was assumed that the transfer length of the bar could vary from a minimum value L_bd,min_ = 256.7 mm, as determined in accordance with Eurocode 2 [18], up to a maximum value L_bd,max_ = 340 mm, which corresponded to the total embedment depth of the bar below the plane of installation of the U-Shaped rebars. The distance between bars and U-shaped rebars must be in the range between 16 and 64 mm, which accounts for the spacing of the steel elements and the other constraints related to their diameters;
- The stresses in the concrete struts were always below 12 MPa. Thus, even when using a low strength concrete class (C20/25, as for the tested specimens), the concrete struts never failed;
- The 3D geometry of the model allowed the definition of the angles between the concrete struts and the steel elements. While Eurocode 2 [18] did not suggest any limit for these angles, lower bound limits should be defined. Indeed, an excessively small angle led to a non-reliable force transfer mechanism between concrete and steel and potential problems for the compatibility of concrete’s deformations. The lower limit of these angles was assumed to be 25° as per ACI 318-14 [35];
- The model shows that the load transfer mechanism from the post-installed bars to the U-shaped rebars could develop properly, regardless of the assumed length of the nodal region t and of the distance between bars and U-shaped rebars.

In summary, for the geometry considered in this application, it seems that the system was able to transfer the applied tension loads from the bars to the U-shaped rebars. This conclusion is clearly applicable only if the U-shaped rebars are anchored properly in the lower portion of the corbel (i.e., bridge deck).

### 4.2. Strut and Tie Model: Anchoring of the U-Shaped Rebars

**Figure 8:**
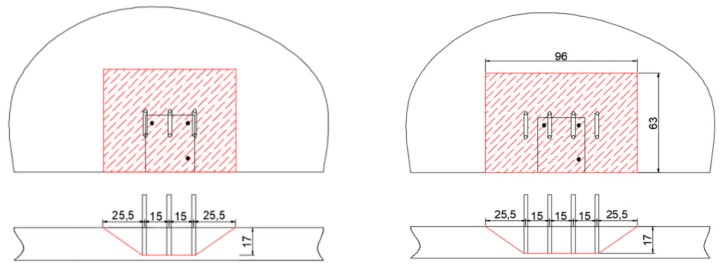
Possible configurations of U-shaped rebars (measures in cm).

The geometry of the anchorage could involve three or four rebars depending on their relative position with respect to the bars (Figure 8). In the following, only the most critical case, which consists in assuming three U-shaped rebars taken as a group of six anchors loaded by a tension load T = 186.64 kN, was considered. Furthermore, it was conservatively assumed that all the six vertical legs of the U-shaped rebars were subjected to the same load, equivalent to the maximum value calculated using the strut and tie model.

**Figure 9:**
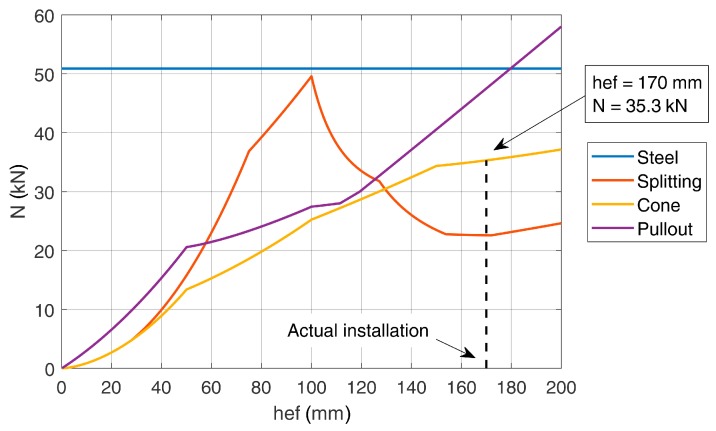
Load carrying capacity as a function of the embedment depth.

Considering a concrete class C20/25 and rebar ∅12 B450C, the classical design verifications (steel, pull-out, concrete cone and splitting failure) can be performed according to [19,23]. The load carrying capacity of the rebar’s leg is shown in Figure 9 for each failure mode as a function of the embedment depth. The splitting failure mode is neglected in this application because reinforcement is always present in bridge decks and can be designed to account for the splitting forces. Figure 9 shows a maximum load carrying capacity for a single leg of the U-shaped rebars equal to 35.29 kN for the proposed embedment of 170 mm. Note that the governing failure mechanism is the concrete cone.

**Figure 10:**
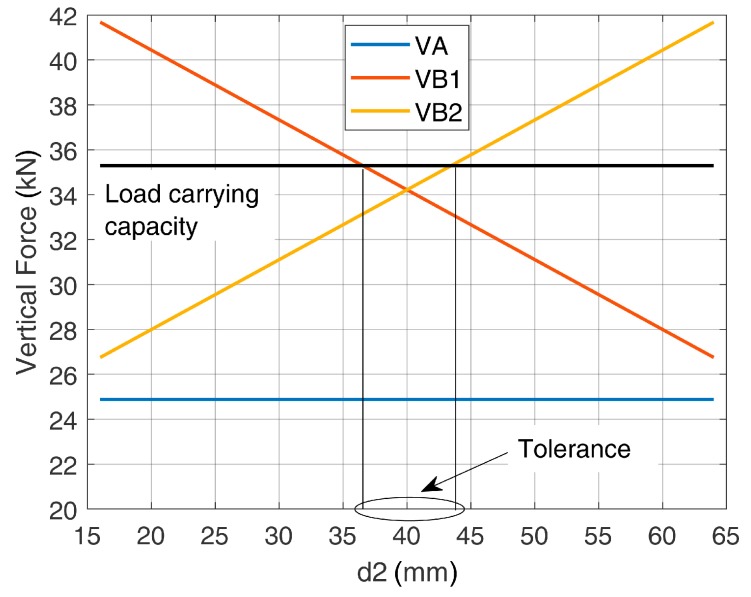
Forces in the vertical legs of the U-shaped rebars and allowed tolerance in the positioning.

When considering the positioning of the rebars and in accordance with the strut and tie modeling, the most favorable case was represented by the symmetric configuration between U-shaped bars and post-installed bars (d_2_ = 40 mm). This configuration led to a tensile force of 34.22 kN on the most stressed element (vertical leg of the central U-shaped rebar). Figure 10 shows that some deviations (<10 mm) from the symmetric configuration were still acceptable, i.e., design verifications were satisfied.

In summary, the strut and tie mechanism developed between the post-installed bars and the U-shaped rebars was able to transfer the applied tensile load of the safety barrier from the bars to the U-shaped rebars, provided that the U-shaped rebars were anchored properly into the concrete slab. An additional optimization of the geometry should be performed to increase the tolerance of the system.

### 4.3. Numerical Analysis

A finite element model of the connection was developed using the software MIDAS FEA [36] to check the accuracy of the results of the strut and tie model. A simplified model considering one bar and two U-shaped rebars and a complete model with the bars, the U-shaped rebars and the cold joint were developed.

The concrete mechanical properties were chosen in accordance with Eurocode 2 [18] for a concrete class C20/25 (elastic modulus E_c_ = 30 GPa and average compressive strength f_cm_ = 28 MPa) and the steel was assumed to behave elastically (elastic modulus E_s_ = 200 GPa). The tensile behavior of the concrete was modeled using a linear total strain crack law characterized by a tensile strength f_ct_ = 2.2 MPa and a fracture energy $G_{f}=73\cdot f_{cm}^{0.18}=132\;N/m$. Perfect bond between the steel and concrete interface was assumed.

The maximum element mesh size of the model ranges between 10 mm for the concrete elements close to the bars (which were modeled using steel beam elements of equivalent size), and 20 mm for the elements far from the bars. These dimensions were chosen on the basis of a mesh-size sensitivity analysis. The geometry of the corbel presents a cold joint between the upper face of the slab and the lower face of the corbel, where the safety barrier is anchored. The bars, as well as the U-shaped rebars, pass through this cold joint. This joint, which is a surface characterized by lower mechanical properties, should be properly modeled to represent the actual behavior of the system. For this reason, a thin layer of concrete elements (about 1 cm in size) was modeled with a reduced tensile strength (1.2 MPa [37]) compared to the typical 2.2 MPa for a C20/25 concrete.

**Figure 11:**
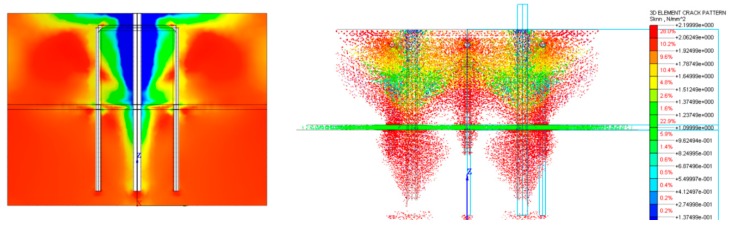
Principal compressive stress field (lateral view-**left**), crack pattern (front view-**right**).

**Figure 12:**
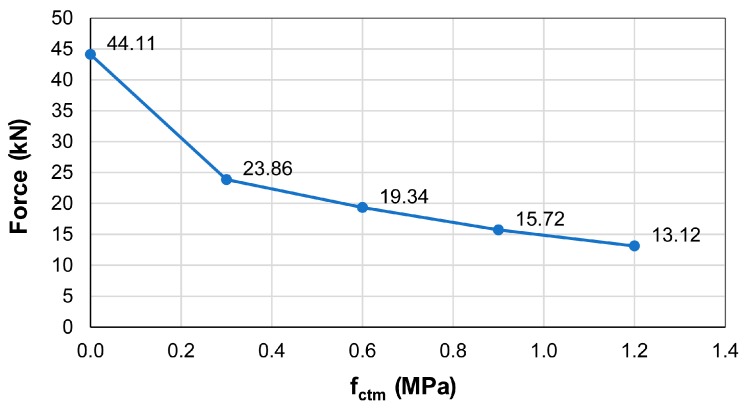
Force transferred by the U-shaped rebar.

The simplified model (1 bar and 2 U-shaped rebars) showed that the addition of the U-shaped rebars led to an increase of the load carrying capacity of the system of about 10%–15%. Figure 11 shows the principal stress field and crack pattern when the overall anchor group behavior (the two bars and the three U-shaped rebars) was considered. As shown in this figure, stresses were transferred from the rebars to the U-shaped bars as schematically represented by the strut and tie model described in Section 4.1. It is noted that the concrete tensile strength f_ct_ of the joint plays a primary role in the overall behavior of the corbel. As shown in Figure 12, in fact, the force transferred from the central U-shaped rebar to the lower portion of concrete slab increased as f_ct_ decreased from 1.2 to 0 MPa.

## 5. Design Optimization

### 5.1. Analytical Approach

The proposed strut and tie model (Section 4.1) can be used to determine the best design configuration of the anchorage system. The previous results highlight that the critical aspect of the connection was the carrying capacity of the U-shaped rebar embedded into the concrete slab. To improve the efficiency of this connection and to increase the tolerance on the location of the rebars (crucial aspect in jobsite), it is possible to perform a parametric investigation to identify the most effective configuration.

**Figure 13:**
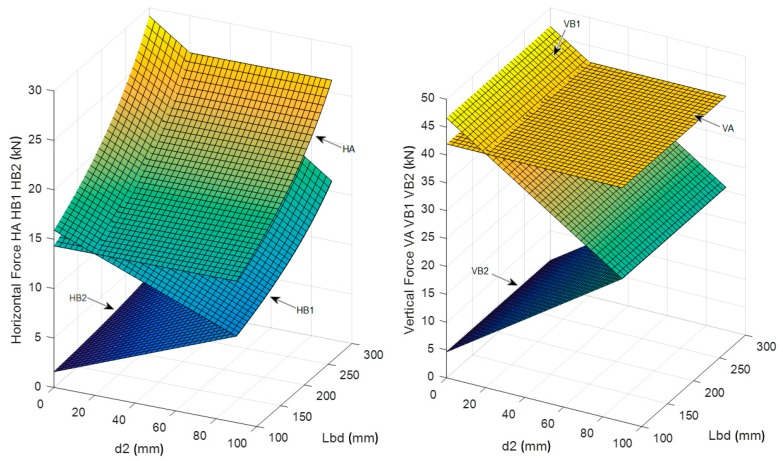
Horizontal and vertical forces in the U-shaped rebars depending on the position d_2_ and on bar transfer length L_bd_ (for: l_m_ = 320 mm, s_2_ =200 mm and t = 60 mm).

To increase the tensile load carrying capacity, the spacing s_2_ between the U-shaped rebars was increased, resulting in a larger area involved in the failure mechanism. The results of the parametric investigation are shown in Figure 13, where the forces acting on the U-shaped rebars are plotted as a function of d_2_ and L_bd_ for selected values of s_2_, l_m_ and t. Note that these values were selected to be representative of typical jobsite conditions. As shown in Figure 13, the resistance of the U-shaped rebar was always sufficient to allow the development of the appropriate load carrying mechanism.

When considering the behavior of the concrete, it seems that the maximum stresses were obtained for the lower values of the assumed anchorage length. 

**Figure 14:**
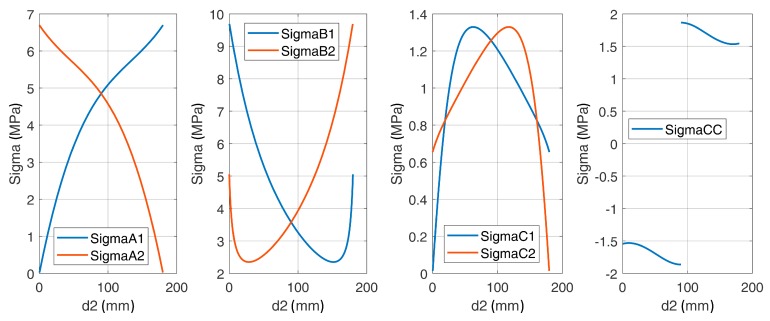
Stresses in the concrete struts as a function of the rebar position.

Nevertheless, even when assuming the minimum bonded length in accordance with [18], the design of the concrete struts was always verified for concrete classes equal to or higher than C20/25, as shown in Figure 14. It is interesting to note that the forces in the vertical legs of the U-shaped rebar did not depend on the assumed transfer length L_bd_ of the bars but rather on the relative position d_2_ between the bars and the U-shaped rebars.

**Figure 15:**
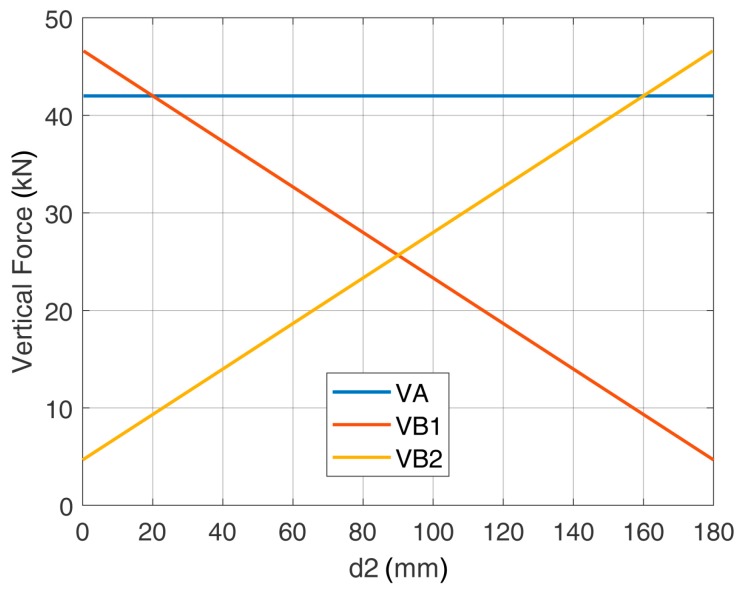
Stresses in the vertical legs of the U-shaped rebars as a function of the relative position.

Figure 15 shows that the minimum resistance that must be guaranteed by the anchorage for each leg of the U-shaped rebar was 42 kN. This value is related to the force in the central U-shaped rebar and ensures a positioning tolerance of ±70 mm from the symmetric case. It is noted that this tolerance is enough to practically cover all the possible installation configurations.

**Figure 16:**
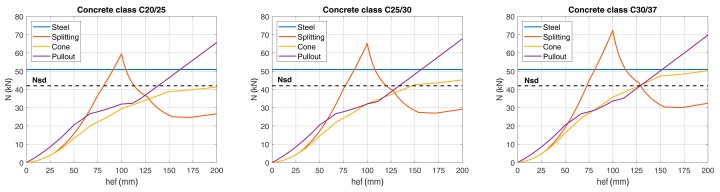
Load carrying capacity of the anchorage for each vertical rebar—different concrete classes.

The embedment depth of the U-shaped rebars can be evaluated as a function of the required resistance of the anchorage in the concrete slab. The verification of the system of six vertical legs of the U-shaped rebars could be performed with commercial software [38] and it was shown that the system was always verified if an embedment depth of 150 mm in the concrete slab was used. Alternatively, by considering the highly stressed leg of the U-shaped rebars (N_Sd_ = 42 kN), the tensile resistance of the connection could be evaluated as a function of the embedment depth for different concrete classes (Figure 16). The load carrying capacity for concrete C20/25 was too low for the considered embedment length of 150 mm. For concrete C25/30 and C30/37, the connection was verified with an embedment depth of 147 mm (total length of the rebar L_m_ > 317 mm) and 129 mm (total length of the rebar L_m_ > 299 mm), respectively. Note that splitting failure was neglected for the reasons previously outlined. When considering this type of failure mode, the design verification would be satisfied only for concrete C30/37 and an embedment depth of 129 mm (L_m_ = 299 mm).

### 5.2. Numerical Approach

The two most extreme arrangements (symmetric and non-symmetric) of the optimized configuration were modeled with nonlinear finite elements, as described above.

**Figure 17:**
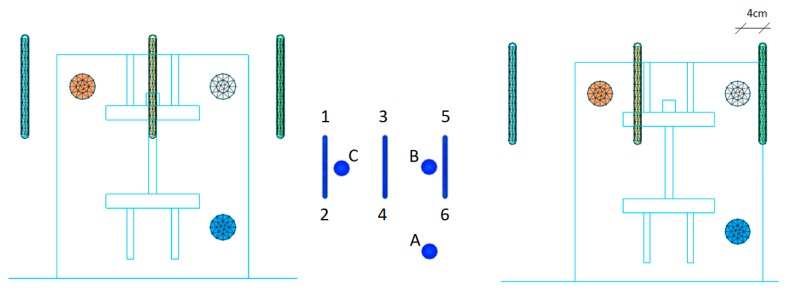
Symmetric configuration (**left**) and asymmetric configuration (**right**).

**Figure 18:**
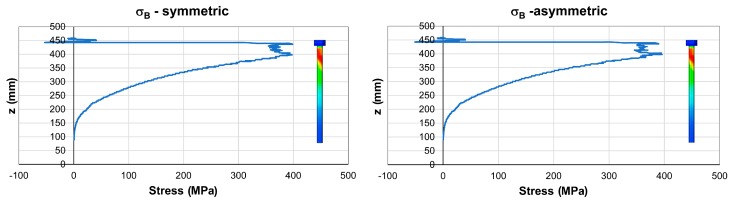
Stresses along bars (MPa)—symmetric (**left**) and asymmetric (**right**) configurations.

**Figure 19:**
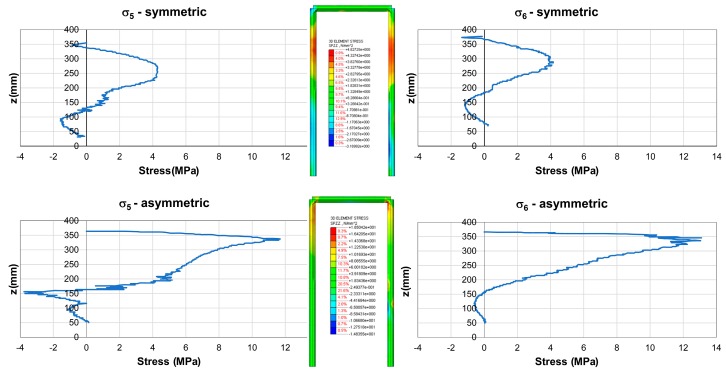
Stresses along the vertical branches of the U-shaped rebars (MPa): σ_5_ (**left**) and σ_6_ (**right**).

The distance between the U-shaped rebars s_2_ was kept equal to 200 mm, with the minimum distance to the closest post-installed bar d_2_ equal to 40 mm for the non-symmetric case, as shown in Figure 17. The stresses along the bar B and in the vertical legs of the U-shaped rebars (5 and 6) are shown in Figure 18 and Figure 19.

As reported in Table 2, it appeared that typical jobsite installations (non-symmetric) were leading to higher stresses. Nevertheless, the design verifications were always verified.

## 6. Conclusions

The installation of safety barriers with the use of three post-installed bars typically does not fulfill the design requirements of the existing standards [1,19,20]. The proposed solution, with the addition of U-shaped rebars, seems to be effective from the experimental, analytical and numerical point of view.

The proposed 3D strut and tie model was able to capture the actual behavior of the connection (as confirmed by numerical analyses) and allowed the determination of the stresses acting on the U-shaped rebars, which are the most stressed elements in the system. Verifications of the concrete struts seem to be always satisfied for commonly adopted concrete classes. The proposed optimized solution demonstrated that by assuming a 200 mm spacing between the U-shaped rebars, their positioning could be performed without tolerance problems.

The proposed user-friendly equations allowed the design verification of the connection with only two steps: (i) steel stresses and (ii) anchorage of the U-shaped rebars into the slab (bridge deck). This fast verification is always on the safe side because it neglects the tensile strength of the concrete. Indeed, the numerical analyses show that the stresses in the U-shaped rebars were dramatically lower when evaluated considering the actual concrete tensile strength.

## Figures and Tables

**Table 1:** Geometrical and mechanical characteristics (see also Figure 2).

Fixture	Bars	U-Shaped Rebars	Materials
b = 300 mm	∅_b_ = 20 mm	∅_r_ = 12 mm	E_c_ = 30 GPa
h = 350 mm	d_b1_ = 200 mm	s_1_ = 150 mm	E_s_ = 210 GPa
s_h_ = 220 mm	d_b2_ = 80 mm	s_2_ = 150 mm	f_yk,rebar_ = 450 MPa
-	h_1_ = 200 mm	d_b3_ = 125 mm	f_yk,bar_ = 500 MPa
-	h_2_ = 200 mm	h_3_ = 30 mm	f_cd_ = 20 MPa
-	s_v_ = 220 mm	h_4_ = 30 mm	ε_c1_ = 2‰
-	b_c_ = 300 mm	h_ef_ = 170 mm	ε_sy_ = 1.956‰
-	l_b_ = 370 mm	l_m_ = 340 mm	-

**Table 2:** Stresses and actions in each vertical leg in the symmetric and asymmetric configuration.

Element	Symmetric		Asymmetric		Difference	
	σ (MPa)	F (kN)	σ (MPa)	F (kN)	σ (MPa)	F (kN)
A	34.66	10.86	33.94	10.66	1.38	0.43
B	401.88	98.64	401.19	98.86	0.69	0.22
C	395.17	95.52	395.03	95.58	0.15	0.06
1	4.31	0.34	2.33	0.18	4.35	0.49
2	3.77	0.29	6.12	0.48	0.92	0.52
3	3.97	0.31	8.78	0.69	4.81	0.54
4	3.97	0.31	5.57	0.44	1.60	0.18
5	4.21	0.33	11.67	0.92	7.46	0.84
6	4.19	0.33	13.05	1.03	8.86	1.00
